# QuickStats

**Published:** 2014-06-27

**Authors:** FROM THE NATIONAL CENTER FOR HEALTH STATISTICS

**Figure:**
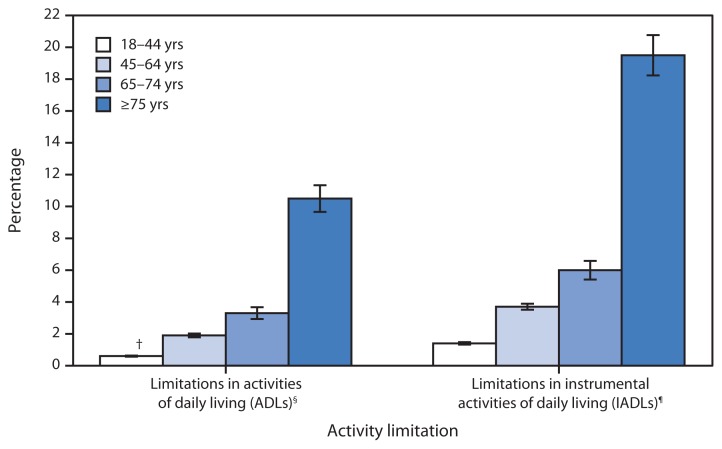
Percentage of Adults with Activity Limitations, by Age Group and Type of Limitation — National Health Interview Survey,* United States, 2012 * Estimates are based on household interviews of a sample of the civilian noninstitutionalized U.S. population. Persons with unknown limitation status were excluded from the denominators. ^†^ 95% confidence interval. ^§^ Limitations in ADLs are based on response to the question, “Because of a physical, mental, or emotional problem, does [person] need the help of other persons with personal care needs, such as eating, bathing, dressing, or getting around inside this home?” Respondents were asked to answer regarding themselves and other family members living in the same household. ^¶^ Limitations in IADLs are based on response to the question, “Because of a physical, mental, or emotional problem, does [person] need the help of other persons in handling routine needs, such as everyday household chores, doing necessary business, shopping, or getting around for other purposes?” Respondents were asked to answer regarding themselves and other family members living in the same household.

In 2012, the percentages of adults with limitations in activities of daily living (ADLs) and limitations in instrumental activities of daily living (IADLs) increased with age. Adults aged ≥75 years were the most likely to require the help of another person with ADLs and with IADLs.

**Source:** Adams PF, Kirzinger WK, Martinez ME. Summary health statistics for the U.S. population: National Health Interview Survey, 2012. Vital Health Stat 2013;10(259). Available at http://www.cdc.gov/nchs/data/series/sr_10/sr10_259.pdf.

**Reported by:** Patricia F. Adams, pfa1@cdc.gov, 301-458-4063; Michael E. Martinez, MPH, MHSA; Whitney K. Kirzinger, MPH.

